# Histological and transcriptome-wide level characteristics of fetal myofiber hyperplasia during the second half of gestation in Texel and Ujumqin sheep

**DOI:** 10.1186/1471-2164-12-411

**Published:** 2011-08-14

**Authors:** Hangxing Ren, Li Li, Hongwei Su, Lingyang Xu, Caihong Wei, Li Zhang, Hongbin Li, Wenzhong Liu, Lixin Du

**Affiliations:** 1 National Center for Molecular Genetics and Breeding of Animal, Institute of Animal Sciences, Chinese Academy of Agricultural Sciences, Beijing, 100193, China; 2 College of Animal Science and Technology, Shanxi Agricultural University, Taigu, Shanxi, 030801, China; 3 College of Animal Science and Technology, Sichuan Agricultural University, Ya'an, Sichuan, 625014, China; 4 Chongqing Academy of Animal Sciences, Chongqing, 402460, China

## Abstract

**Background:**

Whether myofibers increase with a pulsed-wave mode at particular developmental stages or whether they augment evenly across developmental stages in large mammals is unclear. Additionally, the molecular mechanisms of myostatin in myofiber hyperplasia at the fetal stage in sheep remain unknown. Using the first specialized transcriptome-wide sheep oligo DNA microarray and histological methods, we investigated the gene expression profile and histological characteristics of developing fetal ovine longissimus muscle in Texel sheep (high muscle and low fat), as a myostatin model of natural mutation, and Ujumqin sheep (low muscle and high fat). Fetal skeletal muscles were sampled at 70, 85, 100, 120, and 135 d of gestation.

**Results:**

Myofiber number increased sharply with a pulsed-wave mode at certain developmental stages but was not augmented evenly across developmental stages in fetal sheep. The surges in myofiber hyperplasia occurred at 85 and 120 d in Texel sheep, whereas a unique proliferative surge appeared at 100 d in Ujumqin sheep. Analysis of the microarray demonstrated that immune and hematological systems' development and function, lipid metabolism, and cell communication were the biological functions that were most differentially expressed between Texel and Ujumqin sheep during muscle development. Pathways associated with myogenesis and the proliferation of myoblasts, such as calcium signaling, chemokine (C-X-C motif) receptor 4 signaling, and vascular endothelial growth factor signaling, were affected significantly at specific fetal stages, which underpinned fetal myofiber hyperplasia and postnatal muscle hypertrophy. Moreover, we identified some differentially expressed genes between the two breeds that could be potential myostatin targets for further investigation.

**Conclusions:**

Proliferation of myofibers proceeded in a pulsed-wave mode at particular fetal stages in the sheep. The myostatin mutation changed the gene expression pattern in skeletal muscle at a transcriptome-wide level, resulting in variation in myofiber phenotype between Texel and Ujumqin sheep during the second half of gestation. Our findings provide a novel and dynamic description of the effect of myostatin on skeletal muscle development, which contributes to understanding the biology of muscle development in large mammals.

## Background

Texel sheep, a typical "double muscle" breed due to a *GDF8 *mutation [1-3], are now commercially produced throughout the world, with no adverse effects detected by objective assessments of meat quality [4]. However, evidence for an association between g+6723G > A and decreased intramuscular fat and reduced eating quality has been observed [5]. Compared with Texel sheep, indigenous Chinese Ujumqin sheep, with no *GDF8 *mutation [6], are less muscular and have a higher fat content, but they are superior in terms of perceived meat quality. Therefore, these two sheep breeds provide a good natural model for studying muscle and fat development, as well as for identifying myostatin genes.

Prenatal skeletal muscle development is an important determinant of both muscularity and meat quality [7]. In large precocial species such as sheep [8,9] and cattle [10], the maximum myofiber complement of a muscle is achieved prior to birth. More than three waves of myogenic cells appear in sheep, and most myofibers form during the second half of gestation [11,12]. However, whether the myofibers increase with a pulsed-wave mode at certain developmental stages or whether they augment evenly across developmental stages in fetal sheep remains unclear.

Myostatin, a member of the transforming growth factor-β (TGF-β) family, is predominantly expressed and secreted by skeletal muscle and functions as a negative regulator of muscle growth. Mutations in the myostatin gene lead to a hypertrophic phenotype in mice, sheep, cattle, dog, and human [1,3,5,13-18]. The effect of myostatin on gene expression in prenatal muscles at the genome-wide level was recently explored in fetal cattle [19-22], but no studies have been conducted dynamically at multiple fetal stages comparing two pure breeds with extreme phenotypes. A recent mice study demonstrated that myoblasts from embryonic and fetal stages not only had different fusion abilities, proliferation, differentiation and responses to TGF-β, phorbol ester 12-*O*-tetradecanoylphorbol-13-acetate, and bone morphogenetic protein-4 *in vitro*, but they also differed in gene expression profiles [23], indicating that complicated and obvious changes in physiology and biochemistry occur during the prenatal stage *in vivo*. Therefore, investigating the subtle changes of the effect of a myostatin mutation on skeletal muscle development at multiple fetal stages using our experimental model is necessary.

Here, we examined gene expression and myofiber development in Texel and Ujumqin sheep at the transcriptome and histological levels. Our findings contribute to understanding the dynamic effects of myostatin on the biology of prenatal skeletal muscle development in large mammals. The results also provide clues into human myopathy and obesity during prenatal stages. We also identified putative candidate genes that are valuable for meat-quality traits in farm animals.

## Results

### Histological characteristics of ovine fetal skeletal muscle development

**Figure 1:**
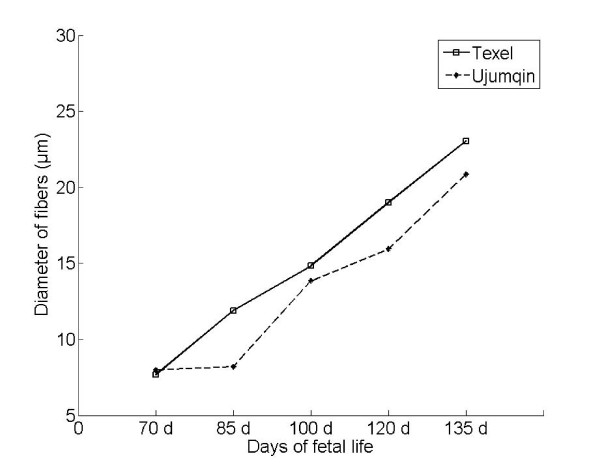
**Changes in muscle fiber diameter throughout the five developmental stages in Texel and Ujumqin sheep**. Longissimus dorsi muscle fiber diameter was examined in cross sections using hematoxylin and eosin staining. At least three animals were used to analyze each development stage in each breed. Photomicrographs of five to eight randomly selected areas were used to calculate the diameter of myofibers in each cross section of muscle examined.

**Figure 2:**
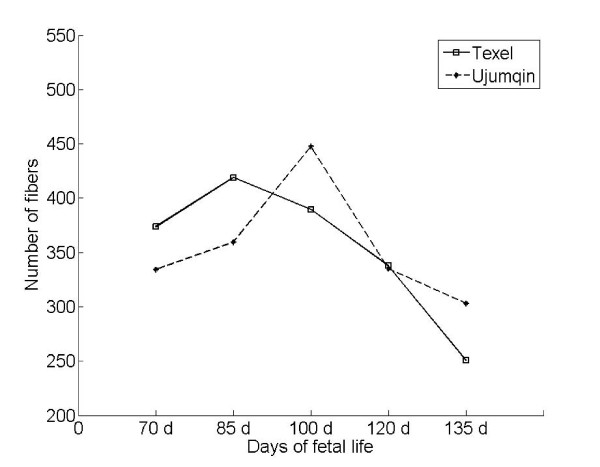
**Changes in muscle fiber number throughout the five developmental stages in Texel and Ujumqin sheep**. Longissimus dorsi muscle fiber number was examined in cross sections using hematoxylin and eosin staining. At least three animals were used to analyze each development stage in each breed. Photomicrographs of five to eight randomly selected areas were used to calculate the total number of myofibers in each cross section of muscle examined.

We examined the number and diameter of myofibers at each developmental stage in Texel and Ujumqin sheep via histological analysis (hematoxylin and eosin [H and E] staining) (Figures 1 and 2). Significant difference was observed in myofiber diameter between Texel and Ujumqin sheep at each development stage, except at 70 d. However, the myofiber diameter patterns of the two breeds were similar throughout the five developmental stages.

Unexpectedly, we found that the myofiber hyperplasia profiles differed between Texel and Ujumqin fetuses (Figure 2). The proliferation of myofibers in Texel sheep was fastest at 70 and 85 d, particularly at 85 d, suggesting that this period is vital for muscle development in fetal Texel sheep. Myofiber hyperplasia reached a peak at 100 d in Ujumqin sheep, when the number of myofibers exceeded that of Texel sheep. Notably, with development, the number of myofibers in Texel fetuses recovered to that of Ujumqin fetuses at 120 d, although myofiber hypertrophy in Texel sheep was greater than that in Ujumqin sheep at this stage. Therefore, a new wave of myogenesis most probably occurs at 120 d in Texel sheep, given that the number of myofibers increased in Ujumqin sheep during this period.

**Figure 3:**
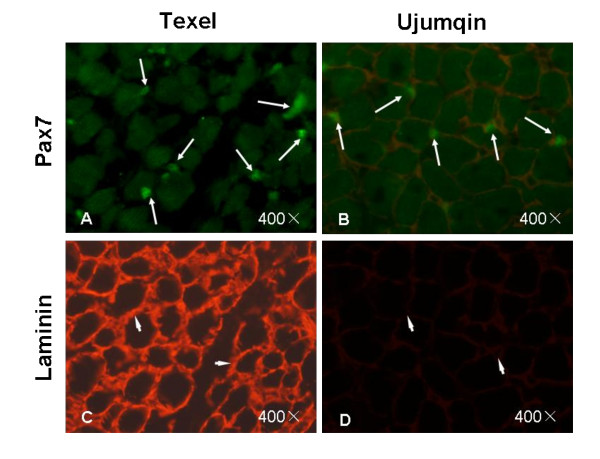
**Identification of Pax7-positive cells in longissimus dorsi muscle by immunofluorescence staining**. Longissimus tissue sections were stained with fluorescent antibodies Pax7 (green) and laminin (red) at 135 d of gestation, respectively. A-D are the same frozen section stained with the two different fluorescent antibodies (400×). The corresponding antigens in muscle are indicated by an arrow (Pax7) and an arrowhead (laminin).

Additionally, many small-diameter fibers occurred in the muscle at 135 d in both breeds. The small fibers could be due either to intrafascicular terminations [24] or have been generated by the fusion of myoblasts during the period investigated. Therefore, to explore the myogenic potential in the two breeds of sheep, we identified Pax7-positive cells in skeletal muscle at 135 d using immnofluorescence staining (IFS) and found that the ratio of the number of Pax7-positive cells (myoblasts or satellite cells) to the fibers on each muscle slide was significantly higher in the Texel fetuses than in the Ujumqin fetuses (Figure 3). In combination with Figure 2, this observation suggests that a period of 100-120 d is another new proliferative stage for myofiber hyperplasia in Texel sheep. Higher numbers of Pax7-positive cells were correlated with a higher muscle mass in Texel sheep during both the prenatal and postnatal stages.

### Microarray experiment

A mean transcript sequence identity of 97% was found between ovine and bovine orthologs, which probably highlights the utility of the microarray technique with ovine samples [25]. However, some no-reporting probe sets were found, partly because of the poor performance of the probe sets with samples from a related species. The best way to resolve this type of problem is to use a species-specific microarray. In the present study, we applied the first specialized and standardized transcriptome-wide sheep oligo DNA microarray (Agilent Sheep Gene Expression Microarray; Agilent Technologies, Santa Clara, CA, USA). Due to improvements in species specificity, the average detectable rate of all probe sets reached 87.83% (Additional file 1), which enabled us to capture the subtle changes in gene expression.

**Figure 4:**
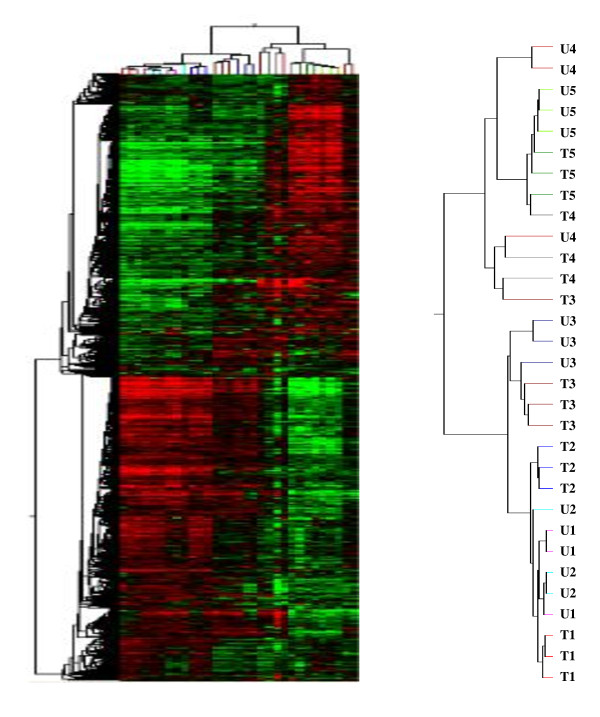
**Hierarchical clustering between the differentially expressed genes and individuals from the two sheep breeds**. Clustering was performed using GeneSpring 10.0. A one-way analysis of variance was applied to two within-breed contrasts across developmental time. A pool of the differentially expressed probes from the two groups was used for system hierarchical clustering to clarify the transcriptome-wide similarities among all 31 individuals investigated.

A pool of differentially expressed (DE) probes from the two groups was used for a systematic hierarchical clustering to gain insight into the transcriptome-wide similarities among all 31 individuals investigated (Figure 4). We found that individuals at the same developmental stage were clustered together regardless of their genetic background, indicating that differences derived during development were larger than those from the genetic backgrounds between the breeds.

### Gene expression in skeletal muscle at various developmental stages between breeds

**Figure 5:**
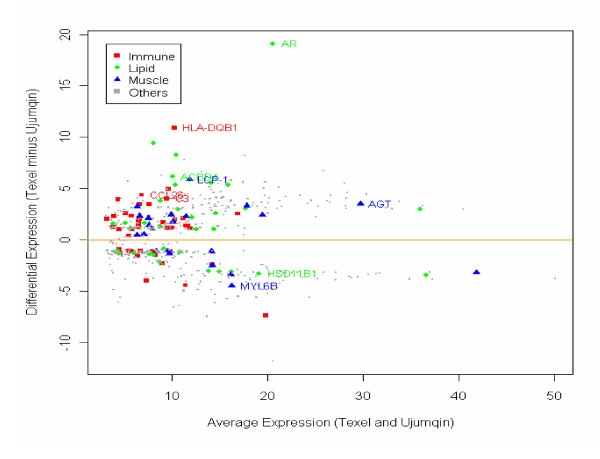
**MA plot for differential genes in Texel and Ujumqin sheep**. Genes expressed more highly in Ujumqin sheep are on the bottom, and genes expressed more highly in Texel sheep are on top. Genes involved in muscles, lipids, and the immune system are denoted by blue triangles, green circles, and red rectangles, respectively. For example, *HLA-DQB1 *and *AR *were highly expressed in Texel, whereas *MYL6B *was highly expressed in Ujumqin sheep. Other genes expressed differentially are denoted by gray rectangles.

**Figure 6:**
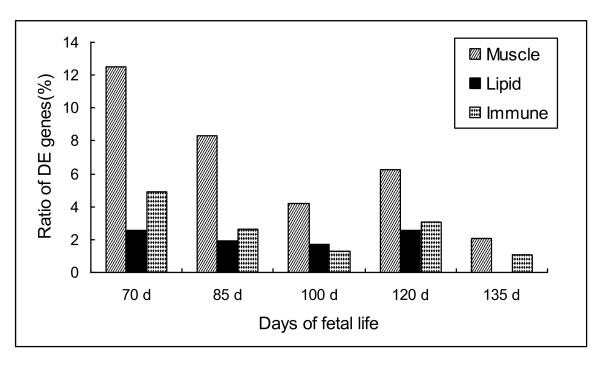
**Abundance of primary functional clustering of differentially expressed genes**. Counts of genes involved in muscles, lipids, and the immune system are represented by the ratio of functional categories relative to the total number of genes that exists in the microarray chip for each of those categories, respectively. The ratio illustrates the abundance of different clusters of differentially expressed genes between Texel and Ujumqin sheep at five developmental stages (70, 85, 100, 120, and 135 d of gestation).

According to the primary functions of different genes including muscle, lipid, and the immune system, we visualized the data on MA and bar plots (Figures 5 and 6). Combined with Figures 1 and 2, Figure 6 also suggests the phenotypic profile in muscle and the profile of a given category of DE genes at different developmental stages. The biological functions and canonical pathways were explored at various developmental stages in Texel and Ujumqin sheep (Tables 1, 2, 3, 4 and 5). The partial DE genes in the two breeds of sheep at various developmental stages are listed in Tables 6 and 7 (the full set of differential genes at each developmental stage between the breeds is listed in Additional files 2, 3, 4, 5 and 6).

**Table 1:** Summary of the differentially expressed genes between T70 and U70 using IPA

Top Bio Functions		
Diseases and Disorders		
Name	p-value^1^	Molecules^2^
*Cance*r^4^	5.82E-08-.93E-03	51
*Cardiovascular Disease*^4^	2.61E-06-7.93E-03	19
Developmental Disorder	2.61E-06-7.93E-03	12
Hypersensitivity Response	8.91E-06-7.93E-03	8
Inflammatory Response	1.05E-05-7.93E-03	25
**Molecular and Cellular Functions**		
**Name**	**p-value**	**Molecules**
Cell-To-Cell Signaling and Interaction	1.77E-06-7.93E-03	32
Cellular Movement	8.91E-06-7.93E-03	25
Lipid Metabolism	9.16E-06-7.93E-03	25
Molecular Transport	9.16E-06-7.93E-03	28
Small Molecule Biochemistry	9.16E-06-7.93E-03	30
**Physiological System Development and Function**		
**Name**	**p-value**	**Molecules**
Tissue Development	1.77E-06-7.93E-03	24
Hematological System Development and Function	7.13E-06-7.93E-03	22
Hematopoiesis	8.91E-06-7.93E-03	9
Immune Cell Trafficking	8.91E-06-7.93E-03	19
Skeletal and Muscular System Development and Function	2.68E-05-7.93E-03	18
**Top Canonical Pathways**		
**Name**	**p-value**	**Ratio^3^**
Acute Phase Response Signaling	2.2E-03	6/178 (0.034)
Glycolysis/Gluconeogenesis	7.65E-03	4/142 (0.028)
*N*-Glycan Biosynthesis	9.44E-03	3/93 (0.032)
Calcium Signaling	1.17E-02	5/204 (0.025)
CXCR4 Signaling	3.31-02	4/169 (0.024)

1. The p-value was calculated using a right-tailed Fisher's exact test. 2. The number of genes enriched in corresponding functions. 3. The ratio was calculated as the number of genes from our data set that overlap with the canonical pathway in question divided by the total number of genes represented in that canonical pathway.

4. Italicized terms such as "*Cancer*" and "*Cardiovascular Disease*" represent a biased annotation or are not related to the aim of the study.

IPA version: 8.7 (release date: 09-17-2010)

Content version: 3202 (release date: 07-07-2010)

Reference set: Ingenuity knowledge base (genes only)

Relationships to include: direct and indirect

Includes endogenous chemicals

Filter summary: Consider all molecules and/or relationships

**Table 2:** Summary of the differentially expressed genes between T85 and U85 using IPA

Top Bio Functions		
Diseases and Disorders		
Name	p-value^1^	Molecules^2^
*Cancer*^4^	2.37E-07-1.98E-02	35
Inflammatory Response	3.15E-06-2.00E-02	23
Developmental Disorder	1.37E-05-1.94E-02	8
Skeletal and Muscular Disorder	5.76E-05-1.85E-02	33
Genetic Disorder	5.77E-05-2.00E-02	54
**Molecular and Cellular Functions**		
**Name**	**p-value**	**Molecules**
Cell-To-Cell Signaling and Interaction	3.46E-05-2.00E-02	23
Lipid Metabolism	2.49E-04-2.00E-02	16
Molecular Transport	2.49E-04-2.00E-02	14
Small Molecule Biochemistry	2.49E-04-2.00E-02	18
*Drug Metabolism*^4^	3.71E-04-2.00E-02	5
**Physiological System Development and Function**		
**Name**	**p-value**	**Molecules**
Hematological System Development and Function	3.46E-05-2.00E-02	17
*Endocrine System Development and Function*^4^	3.71E-04-1.98E-02	5
*Visual System Development and Function*^4^	3.71E-04-3.71E-04	2
Hematopoiesis	3.72E-04-2.00E-02	8
Immune Cell Trafficking	3.72E-04-1.98E-02	16
**Top Canonical Pathways**		
**Name**	**p-value**	**Ratio^3^**
Glycolysis/Gluconeogenesis	1.34E-04	5/142 (0.035)
Fructose and Mannose Metabolism	2.19E-04	4/145 (0.028)
Pentose Phosphate Pathway	9.13E-04	3/89 (0.034)
Dendritic Cell Maturation	1.31E-03	5/188 (0.027)
VEGF Signaling	9.55E-03	3/99 (0.03)

Symbols and analytical settings are the same as in Table 1.

**Table 3:** Summary of differentially expressed genes between T100 and U100 using IPA

Top Bio Functions		
Diseases and Disorders		
Name	p-value^1^	Molecules^2^
*Respiratory Disease*^4^	7.71E-05-2.60E-02	3
Metabolic Disease	1.05E-04-2.51E-02	8
Hypersensitivity Response	2.08E-04-2.51E-02	3
Inflammatory Response	2.08E-04-3.57E-02	11
*Cancer*^4^	5.03E-04-1.92E-02	14
**Molecular and Cellular Functions**		
**Name**	**p-value**	**Molecules**
Lipid Metabolism	2.91E-11-3.57E-02	16
Small Molecule Biochemistry	2.91E-11-3.57E-02	21
Vitamin and Mineral Metabolism	2.91E-11-2.60E-02	16
Molecular Transport	8.41E-06-3.57E-02	19
Cellular Movement	2.08E-04-3.57E-02	5
**Physiological System Development and Function**		
**Name**	**p-value**	**Molecules**
Hematological System Development and Function	2.08E-04-3.57E-02	11
Immune Cell Trafficking	2.08E-04-3.57E-02	9
Lymphoid Tissue Structure and Development	2.08E-04-3.57E-02	4
Tissue Development	4.79E-04-3.57E-02	9
Hematopoiesis	6.96E-04-3.57E-02	6
**Top Canonical Pathways**		
**Name**	**p-value**	**Ratio^3^**
T-Helper Cell Differentiation	2E-03	3/72 (0.042)
Inositol Metabolism	3.73E-03	3/97 (0.031)
Biosynthesis of Steroids	5.98E-03	2/128 (0.016)
EIF2 Signaling	3.89E-02	2/103(0.019)
VEGF Signaling	3.98E-02	2/99 (0.02)

Symbols and analytical settings are the same as in Table 1.

**Table 4:** Summary of differentially expressed genes between T120 and U120 using IPA

Top Bio Functions		
Diseases and Disorders		
Name	p-value^1^	Molecules^2^
Hypersensitivity Response	9.54E-06-1.19E-02	7
Inflammatory Response	9.54E-06-1.19E-02	18
*Cancer*^4^	3.78E-05-1.19E-02	41
Hematological Disease	5.66E-05-1.19E-02	7
Immunological Disease	5.66E-05-1.19E-02	7
**Molecular and Cellular Functions**		
**Name**	**p-value**	**Molecules**
Cell-To-Cell Signaling and Interaction	9.54E-06-1.19E-02	17
Lipid Metabolism	1.16E-05-1.19E-02	16
Small Molecule Biochemistry	1.16E-05-1.19E-02	20
Carbohydrate Metabolism	3.54E-05-1.19E-02	5
*Drug Metabolism*^4^	3.54E-05-1.19E-02	10
**Physiological System Development and Function**		
**Name**	**p-value**	**Molecules**
Hematological System Development and Function	9.54E-06-1.19E-02	23
Immune Cell Trafficking	9.54E-06-1.19E-02	13
Nervous System Development and Function	1.69E-05-1.19E-02	8
Tissue Development	1.69E-05-1.19E-02	13
*Reproductive System Development and Function*^4^	7.17E-05-1.19E-02	10
**Top Canonical Pathways**		
**Name**	**p-value**	**Ratio^3^**
*Altered T Cell and B Cell Signaling in Rheumatoid Arthritis*^4^	1.54E-04	5/91 (0.055)
NF-kB Signaling	1.87E-03	5/155 (0.032)
Autoimmune Thyroid Disease Signaling	2.77E-03	3/61 (0.049)
*Atherosclerosis Signaling*^4^	2.92E-03	4/113 (0.035)
Taurine and Hypotaurine Metabolism	3.53E-03	2/47 (0.043)

Symbols and analytical settings are the same as in Table 1.

**Table 5:** Summary of differentially expressed genes between T135 and U135 using IPA

Top Bio Functions		
Diseases and Disorders		
Name	p-value^1^	Molecules^2^
*Cancer*^4^	9.23E-06-2.87E-02	7
*Reproductive System Disease*^4^	9.23E-06-6.44E-03	7
Genetic Disorder	3.22E-03-2.87E-02	5
Immunological Disease	3.22E-03-3.22E-03	1
Infection Mechanism	3.22E-03-4.11E-02	4
**Molecular and Cellular Functions**		
**Name**	**p-value**	**Molecules**
Cell Cycle	5.50E-04-2.87E-02	3
Cell Death	1.04E-03-2.43E-02	8
Cell-To-Cell Signaling and Interaction	3.22E-03-4.42E-02	5
Cellular Assembly and Organization	3.22E-03-4.42E-02	7
Cellular Compromise	3.22E-03-4.42E-02	3
**Physiological System Development and Function**		
**Name**	**p-value**	**Molecules**
Embryonic Development	5.50E-04-2.55E-02	5
Tissue Development	5.50E-04-4.11E-02	7
Hematological System Development and Function	2.47E-03-4.42E-02	7
Cell-mediated Immune Response	3.22E-03-4.42E-02	2
Nervous System Development and Function	3.22E-03-6.44E-03	2
**Top Canonical Pathways**		
**Name**	**p-value**	**Ratio^3^**
LPS/IL-1-mediated Inhibition of RXR Function	3.61E-03	4/216 (0.019)
Histidine Metabolism	1.26E-02	2/120 (0.017)
PXR/RXR Activation	2.2E-02	2/91 (0.022)
Arginine and Proline Metabolism	2.68E-02	2/183 (0.011)
Glycerolipid Metabolism	4.51E-02	2/156 (0.013)

Symbols and analytical settings are the same as in Table 1.

**Table 6:** Examples of downregulated genes between Texel and Ujumqin sheep at the same developmental stage

Probe Name	Genbank Accession Number	Gene	Fold Change	Developmental stage
A_70_P069356	EE752033	*Homo sapiens *selenoprotein I (SELI)	15.35	70 d
A_70_P016291	EE793140	*Homo sapiens *Rho-related BTB domain containing 3 (RHOBTB3)	5.16	70 d
A_70_P006561	EE781286	*Homo sapiens *nucleolar protein 7 (NOL7)	4.54	70 d
A_70_P004621	EE863207	*Homo sapiens *signal transducer and activator of transcription 3 (STAT3)	2.38	70 d
A_70_P001706	FE033261	*Homo sapiens *aldolase A, fructose-bisphosphate, transcript variant 1 (ALDOA)	2.28	70 d
A_70_P056812	NM_001009763	*Homo sapiens *leptin receptor (LEPR)	2.24	70 d
A_70_P005526	CO202686	*Homo sapiens *stathmin-like 2 (STMN2)	8.23	85 d
A_70_P016291	EE793140	*Homo sapiens *Rho-related BTB domain containing 3 (RHOBTB3)	5.99	85 d
A_70_P068196	EE779349	*Homo sapiens *trefoil factor 3 (TFF3)	5.19	85 d
A_70_P039551	NM_001009266	*Homo sapiens *proopiomelanocortin (POMC)	5.00	85 d
A_70_P037096	EE773353	*Homo sapiens *phosphoinositide-3-kinase, regulatory subunit 1 (alpha) (PIK3R1), transcript variant 2	2.77	85 d
A_70_P001731	EE780294	*Homo sapiens *trafficking protein, kinesin-binding 1 (TRAK1)	8.04	100 d
A_70_P017916	EE828626	*Homo sapiens *S100 calcium-binding protein A11(S100A11)	8.03	100 d
A_70_P032741	CF116713	*Bos taurus *defensin, beta (DEFB)	7.09	100 d
A_70_P001411	EE873259	*Bos taurus *secretoglobin, family 1D, member 2 (SCGB1D2)	5.59	100 d
A_70_P039326	NM_001009410	*Homo sapiens *thyroid-stimulating hormone receptor (TSHR)	4.50	100 d
A_70_P045271	EE788300	*Homo sapiens *paraoxonase 1 (PON1)	4.01	100 d
A_70_P068131	DQ152973	*Homo sapiens *lymphoid enhancer-binding factor1 (LEF-1)	2.23	100 d
A_70_P035741	FE033512	*Bos taurus *X (inactive)-specific transcript (XIST), noncoding RNA	65.59	120 d
A_70_P005526	CO202686	*Homo sapiens *stathmin-like 2 (STMN2)	21.27	120 d
A_70_P018856	EE791277	*Homo sapiens *ATP-binding cassette, subfamily B (MDR/TAP), member 4 (ABCB4)	15.37	120 d
A_70_P024811	EE777383	*Homo sapiens *epithelial cell adhesion molecule (EPCAM)	7.16	120 d
A_70_P019481	EE828813	*Homo sapiens *transformation/transcription domain-associated protein (TRRAP)	4.92	120 d
A_70_P030147	NM_001009261	*Homo sapiens *prion protein 2 (doublet) (PRND)	4.84	120 d
A_70_P025536	FE022583	*Homo sapiens *glutamate receptor, metabotropic 8 (GRM8)	4.62	120 d
A_70_P051317	NM_001009479	*Homo sapiens *natriuretic peptide precursor C (NPPC)	4.46	120 d
A_70_P030402	NM_001093787	*Homo sapiens *kallikrein 1 human (KLK1)	4.37	120 d
A_70_P051037	NM_001009451	*Homo sapiens *solute carrier family 2 (facilitated glucose/fructose transporter), member 5 (SLC2A5)	8.14	135 d
A_70_P035536	DY520412	*Homo sapiens *ankyrin repeat domain 1 (cardiac muscle) (ANKRD1)	5.37	135 d
A_70_P016291	EE793140	*Homo sapiens *Rho-related BTB domain containing 3 (RHOBTB3)	4.14	135 d

Differentially expressed probes were detected from five *t*-test analyses using GeneSpring 10.0 (Agilent Technologies).

1. Original probe name in microarray analysis. 2. GenBank Accession Number of expressed sequence tags provided by the manufacturer according to the specific probe name. 3. The highly homologous human sequences acquired using BLASTN search in NCBI (search setting: query coverage not less than 50%, E-value less than 1.00E-100).

**Table 7:** Examples of upregulated genes between Texel and Ujumqin sheep at the same developmental stage

Probe Name^1^	GenBank^2 ^Accession Number	Gene^3^	Fold Change	Developmental Stage
A_70_P046666	EE795873	*Homo sapiens *eukaryotic translation elongation factor 1 alpha 2 (EEF1A2)	8.92	70 d
A_70_P011966	FE031658	*Homo sapiens *transmembrane channel-like protein 5 (TMC5)	6.74	70 d
A_70_P019721	EE787688	*Homo sapiens *complement component 4-binding protein, alpha (C4BPA)	6.64	70 d
A_70_P024986	EE825499	*Homo sapiens *CD8a molecule, complete cds (CD8A)	6.06	70 d
A_70_P003451	CU652121	*Homo sapiens *cardiomyopathy-associated 5 (CMYA5)	4.09	70 d
A_70_P046666	EE795873	*Homo sapiens *eukaryotic translation elongation factor 1 alpha 2 (EEF1A2)	16.21	85 d
A_70_P063416	EE775784	*Homo sapiens *fructose-1,6-bisphosphatase 2 (FBP2)	10.39	85 d
A_70_P037371	EE776937	*Homo sapiens *actinin, alpha 3 (ACTN3)	7.83	85 d
A_70_P054756	EE791183	*Homo sapiens *apolipoprotein C-II (APOC2)	6.29	85 d
A_70_P055056	EE768552	*Homo sapiens *proteoglycan 3 (PRG3)	6.03	85 d
A_70_P050846	CU651713	*Homo sapiens *myosin, heavy chain 1, skeletal muscle, adult (MYH1)	5.60	85 d
A_70_P019721	EE787688	*Homo sapiens *complement component 4-binding protein, alpha (C4BPA)	4.00	85 d
A_70_P017031	L08792	*Homo sapiens *major histocompatibility complex, class II, DQ beta 1 (HLA-DQB1)	131.77	100 d
A_70_P044801	EE862627	*Homo sapiens *chemokine ligand 26-like protein (CCL26)	10.16	100 d
A_70_P024246	EE859238	*Homo sapiens *pyruvate dehydrogenase kinase, isozyme 4 (PDK4)	5.85	100 d
A_70_P057421	EE784201	*Homo sapiens *MAK16 homolog (*S. cerevisiae*) (MAK16)	5.22	100 d
A_70_P070736	EE824836	*Homo sapiens *thioredoxin reductase mRNA (TXNRD1)	5.20	100 d
A_70_P011371	EE813532	*Homo sapiens *isopentenyl-diphosphate delta isomerase 1 (IDI1)	4.25	100 d
A_70_P017031	L08792	*Homo sapiens *major histocompatibility complex, class II, DQ beta 1 (HLA-DQB1)	1389.16	120 d
A_70_P066011	FE028749	*Homo sapiens *ubiquitin-specific peptidase 9, Y-linked (USP9Y)	670.44	120 d
A_70_P066386	EE851120	*Homo sapiens *guanylate-binding protein 2, interferon-inducible (GBP2)	76.81	120 d
A_70_P052621	EE778111	*Homo sapiens *pyrophosphatase (inorganic) 1 (PPA1)	14.06	120 d
A_70_P065251	EE748701	*Homo sapiens *CD48 molecule (CD48)	9.68	120 d
A_70_P044801	EE862627	*Homo sapiens *chemokine ligand 26-like protein (CCL26)	7.26	120 d
A_70_P035891	EE805356	*Homo sapiens *sorting nexin 10 (SNX10)	5.93	120 d
A_70_P000126	EE807554	*Homo sapiens *guanylate-binding protein 4 (GBP4)	4.97	120 d
A_70_P016471	EE829459	*Homo sapiens N*-myc upstream-regulated 1 (NDRG1)	4.40	120 d
A_70_P039346	NM_001009789	*Homo sapiens *prostaglandin F receptor (FP) (PTGFR)	4.32	120 d
A_70_P011531	EE776172	*Homo sapiens *CCAAT/enhancer-binding protein (C/EBP), beta (CEBPB)	4.14	120 d
A_70_P045436	EE827672	*Homo sapiens *glutathione *S*-transferase theta 1 (GSTT1)	4.13	120 d
A_70_P025036	EE746884	*Homo sapiens *regulator of G-protein signaling 1 (RGS1)	11.22	135 d
A_70_P049266	EE815004	*Bos taurus *mRNA for T-cell receptor alpha chain, leader sequence, variable and joining region (TRA2)	7.06	135 d
A_70_P030156	NM_001009735	*Homo sapiens *ISG15 ubiquitin-like modifier (ISG15)	4.28	135 d
A_70_P030132	NM_001104930	*Homo sapiens *receptor (chemosensory) transporter protein 4 (RTP4)	4.20	135 d
A_70_P001016	FE027322	*Homo sapiens *eukaryotic translation initiation factor 2, subunit 2 beta (EIF2S2)	3.52	135 d

Differentially expressed probes were detected from five *t*-test analyses using GeneSpring 10.0 (Agilent Technologies).

1. Original probe name in the microarray analysis. 2. The GenBank Accession Number of expressed sequence tags provided by the manufacturer according to the specific probe name. 3. The highly homologous human sequences acquired by a BLASTN search in NCBI (search setting: query coverage not less than 50%, E-value less than 1.00E-100).

#### T70 vs. U70

The rapid increase in total muscle fiber number in the ovine fetus begins at 70 d [11,12]. We found that the number of DE genes was highest at this stage compared with other stages: 207 probe sets, including 155 known genes and 50 expressed sequence tags (ESTs) were differentially expressed in the T70 and U70 samples. Compared with U70, 82 genes were upregulated and 73 genes were downregulated in T70. A gene ontology analysis by DAVID revealed that these upregulated genes encoded cell activation, negative regulation of apoptosis, positive regulation of peptidyl-tyrosine phosphorylation, and vesicle-mediated transport. Genes that were downregulated encoded hexose catabolism, monosaccharide catabolism, postembryonic development, alcohol catabolism, and lipid biosynthesis.

To ascertain whether some molecular events or cascades were associated with the process of skeletal muscle development, we identified four canonical pathways by DAVID and the IPA platforms, which are involved in myogenesis and muscle metabolism, including the glycolysis/gluconeogenesis pathway, *N*-glycan biosynthesis, calcium signaling, and chemokine (C-X-C motif) receptor 4 (CXCR4) signaling).

We also revealed through IPA software that genes associated with the skeletal and muscular system development and function, as well as lipid metabolism, were top biological functions (Table 1).

We further analyzed some transcripts and found that eukaryotic translation elongation factor 1 alpha 2 (*EEF1A2*) was highly expressed in T70. In contrast, the expression levels of signal transducer and activator of transcription 3 (*STAT3*), aldolase A (*ALDOA*), and the leptin receptor (*LEPR*) were upregulated in U70.

#### T85 vs. U85

In total, 115 probe sets were differentially expressed in T85 and U85, including 97 annotated genes and 18 ESTs. In contrast to U85, 50 genes were upregulated and 47 were downregulated in T85. Of these genes, immune response, cellular carbohydrate catabolism, and fatty acid metabolism were significantly overrepresented in the upregulated genes, whereas cell migration regulation was significantly overrepresented in the downregulated genes.

For all DE genes between U85 and T85, we identified four canonical pathways that were overrepresented between the two breeds: glycolysis/gluconeogenesis, fructose and mannose metabolism, the pentose phosphate pathway, and vascular endothelial growth factor (VEGF) signaling. Moreover, hematological system development and function and lipid metabolism were still significantly represented as top biofunctions. In particular, we found a cluster of genes enriched in the biofunction of skeletal and muscular disorders, suggesting a difference in skeletal muscle development between Texel and Ujumqin fetal sheep (Table 2).

Among the DE genes, eukaryotic translation elongation factor 1 alpha 2 (*EEF1A2*), fructose-1,6-bisphosphatase 2 (*FBP2*), actinin alpha 3, proteoglycan 3 (*PRG3*), myosin heavy chain 1 (*MYH1*) were more highly expressed in Texel than in Ujumqin sheep. However, the stathmin-like 2 (*STMN2*) and phosphoinositide-3-kinase, regulatory subunit 1 alpha (*PIK3R1*) were more highly expressed in U85.

#### T100 vs. U100

At this fetal developmental stage, the difference in muscle development, particularly the rate of myofiber proliferation, was most significant between the breeds. Ninety probe sets, including 70 annotated genes and 20 ESTs, were differentially expressed in T100 and U100. Among these genes, 33 were upregulated and 37 were downregulated in T100. The upregulated genes were involved in DNA binding, RNA binding, and ribonucleoproteins, whereas those that were downregulated were mostly related to steroid biosynthesis, lipid metabolism, and skeletal muscle fiber development.

For all DE genes at 100 d, hematological system development and function remained the most overrepresented physiological system, and lipid metabolism was also more active at this stage. Inositol metabolism, steroid biosynthesis, eukaryotic initiation factor 2 (EIF2) signaling, and VEGF signaling were overrepresented between T100 and U100 (Table 3).

Within the DE genes, thyroid stimulating hormone receptor (*TSHR*) and paraoxonase 1 (*PON1*) were more highly expressed in Ujumqin sheep than in Texel sheep, whereas *OVAR-DQB1 *(sheep MHC class II), pyruvate dehydrogenase kinase, isozyme 4 (*PDK4*), and thioredoxin reductase (*TXNRD1*) were upregulated in T100.

#### T120 vs. U120

The discrepancy in fetal muscle development was more significant between Texel and Ujumqin sheep at 120 d. In total, 151 probe sets, including 120 annotated genes and 31 ESTs, were differentially expressed in T120 and U120. Of these genes, 63 were upregulated in T120 and 57 were upregulated in U120. The upregulated genes were specific to the immune response, leukocyte activation, and the I-kappaB kinase/NF-kappaB cascade, whereas the downregulated genes were significantly associated with the response to ethanol, the enzyme-linked receptor protein signaling pathway, and histone H2A acetylation.

Hematological system development and function, immune cell trafficking, and nervous system development and function were most overrepresented in physiological systems development and function. Furthermore, lipid metabolism was also the most overrepresented molecular and cellular function. Several canonical pathways were associated with immune and lipid metabolism disorders, such as nuclear factor (NF)-kB signaling and atherosclerosis signaling (Table 4).

Among DE genes between T120 and U120, *OVAR-DQB1 *(sheep MHC class II), guanylate-binding protein 2; interferon-inducible, guanylate-binding protein 4; and CCAAT/enhancer-binding protein (C/EBP) beta (*CEBPB*) were upregulated in T120. Both *STMN2 *and transformation/transcription domain-associated protein (*TRRAP*) were upregulated in U120.

#### T135 vs. U135

Seventy-two probe sets, including 58 annotated genes and 14 ESTs, were differentially expressed in T135 and U135. Of these genes, 27 were upregulated in T135 and another 31 were more highly expressed in U135. The immune response process was overrepresented in the upregulated genes, and mitosis was represented significantly in the downregulated genes.

For the pool of DE genes, embryonic development and tissue development, hematological system development and function, cell-mediated immune response, and nervous system development and function were listed at the top of physiological system development and function. Cellular functions such as the cell cycle, cell death, and cell-to-cell signaling and interaction were more active. Notably, a pathway involved in glycerolipid metabolism was identified between T135 and U135 (Table 5), two members of which (*ALDH1A1 *and *APOC2*) were upregulated in U135.

In terms of physiological system development and functions, differences in the immune and hematological systems were most significant between Texlel and Ujumqin sheep during muscle development. Then, differences in nervous system development and function became more prominent at later gestational stages. The most obvious differences in molecular and cellular functions were lipid metabolism and cell communication between the two breeds. Several overrepresented canonical pathways related to muscle and adipose development (Table 5), which were particularly noteworthy, underpinned the differences in skeletal muscle and adipose tissue between the two breeds at the fetal stage.

### Quantitative PCR validation of microarray gene expression

To confirm the differential expression of the genes in the microarray analysis, we selected 15 genes to validate by quantitative PCR (qPCR). Among these, eight genes (*ACTB, TNC, MYO9A, MYL6B, MYH1, CASQ1, TPM2*, and *TMOD4*) encode myofibrillar proteins and two (*EEF1A2 *and *LEF-1*) are transcription factors that regulate the genes associated with myogenesis. *PIK3R1 *is involved in fat metabolism regulation, whereas *TRRAP, TXNRD1*, and *INSIG1 *participate in the regulation of gene expression and cell proliferation. Additionally, we also detected *MSTN *expression during skeletal muscle development. As in previous studies [26-29], *RpLP0 *was chosen as an ideal reference gene to normalize the data. The ratios of 12 DE genes at particular developmental stages in the two sheep breeds in the microarray analysis agreed with the qPCR results (Table 8).

**Table 8:** Validation of differential expression by quantitative PCR (qPCR)

		Ratio of gene expression between two sheep breeds^1^				
Gene	Method	T70/U70	T85/U85	T100/U100	T120/U120	T135/U135
*EEF1A2*	qPCR	0.23	1.36	4.17	0.57	0.11
	microarray	8.92	16.21*	1.78	0.78	0.98
*TXNRD1*	qPCR	131.73	382.54	35.06	2.3	1.16
	microarray	1.38	1.14	5.20*	3.15	0.94
*INSIG1*	qPCR	0.82	1.65	0.32	0.16	0.10
	microarray	1.10	1.11	0.34*	0.88	0.87
*LEF-1*	qPCR	6.64	21.65	0.51	0.78	0.16
	microarray	0.52	0.59	0.47*	0.50	0.57
*TMOD4*	qPCR	81.5	714.32	323.03	3.7	0.02
	microarray	2.80*	3.52*	1.54	1.15	0.80
*TPM2*	qPCR	0.29	0.87	1.48	0.17	0.84
	microarray	0.48*	0.60	1.54	3.03	1.10
*MYO9A*	qPCR	0.14	0.15	0.25	0.31	0.12
	microarray	0.49*	0.61	1.02	0.75	1.20
*TRRAP*	qPCR	0.06	0.30	0.85	1.11	0.42
	microarray	0.51	0.35	1.79	0.20*	0.26
*TNC*	qPCR	0.21	0.54	5.12	6.23	10.46
	microarray	0.88	0.69	0.41*	0.62	1.78
*PIK3R1*	qPCR	0.61	0.44	0.23	1.06	0.29
	microarray	0.51	0.36*	1.03	1.92	1.14
*ACTB*	qPCR	1.79	0.16	0.73	0.35	0.85
	microarray	1.09	0.55	0.48*	1.39	0.47
*MYL6B*	qPCR	0.35	1.0	3.44	0.84	0.85
	microarray	0.42*	1.14	0.88	1.16	1.04
*MYH1*	qPCR	0.84	1.36	0.21	0.57	0.56
	microarray	1.89	5.60*	1.56	1.19	1.09
*CASQ1*	qPCR	0.51	0.76	0.56	0.07	2.79
	microarray	2.16 *	2.31	1.23	0.82	0.72
*MSTN*	qPCR	0.81	0.71	0.54	0.47	0.61
	microarray	0.69	0.92	0.62	0.56	0.81

1. The data in the rows are the ratio of the 2^ΔΔCT ^values for the qPCR in a Texel sheep sample relative to that in the Ujumqin sheep sample at the same developmental stage. The microarray row provides gene expression ratios in Texel sheep samples divided by those in Ujumqin sheep samples at the same developmental stage. *Differentially expressed genes in the microarray analysis with a statistically significant difference at p < 0.05.

## Discussion

### Characteristics of prenatal muscle fiber development in sheep

Wilson *et al. *and Maier *et al. *showed that at least three waves of myogenesis occurred until d 76 of gestation in sheep [9,11]. We demonstrated that the myofiber numbers increase sharply with a pulsed-wave mode at a particular developmental stage in sheep. This is the first time that the mode of fetal myofiber proliferation was revealed in a large mammal during the second half of gestation. In addition to the longissimus dorsi muscle, similar patterns of myofiber proliferation were found in the semitendenosus, gluteus medius, gastrocnemius, and triceps brachii muscles in Texel and Ujumqin fetal sheep (data not shown). These findings indicate that the specific developmental stage during which myogenic surges of myofiber hyperplasia appeared is pivotal for fetal muscle development as well as postnatal muscularity potential in sheep.

Unlike the studies of Wilson *et al. *and Maier *et al.*, who identified different generations of myogenic cells using electron microscopy [9,11], we counted the muscle fibers under a light microscope and found many small-diameter fibers until 135 d in both breeds. We considered that at least part of the small fibers must have been generated by the fusion of myoblasts (or satellite cells) during the study period. Pax7 plays an important role conferring myogenic potential to these progenitor cells [30], and Pax7 is an ideal cell marker of myogenic progenitor cells such as myoblasts and satellite cells [31]. Our findings indicate a higher potential for postnatal muscle growth that entails more Pax7-positive cells in the fetal stage.

### Gene expression in the two breeds at various developmental stages

Of the five prenatal stages, the numbers of DE genes in the two sheep breeds were highest at 70 and 120 d, indicating that rapid myofiber proliferation included numerous genes during the myogenic process. However, two surges of myofiber proliferation appeared at 85 and 120 d in Texel fetuses, probably because the expression of more genes associated with myoblast proliferation and differentiation at 70 d prepared for the myofiber hyperplasia that followed at 85 d, and more genes related to the immune system were differentially expressed between T70 and U70 (Figure 6), which led to larger total counts of DE genes. The difference between the two sheep breeds in the timing of myogenesis in terms of transcriptomic levels was similar to that found between pig breeds [32]. Cagnazzo *et al. *showed that myogenesis-related gene expression is greater in early Duroc (a breed with more intramuscular fat) embryos than in early Pietrain (a highly muscled breed) embryos at 14-49 d of gestation, whereas the opposite was found in late embryos (63-91 d of gestation) [32]. The present findings suggest that highly muscled breeds have a longer myogenic process during prenatal stages and that myogenesis is more intense in late-stage fetuses, which was validated by Pax7-cell staining in longissimus muscle cross sections (Figure 3). However, whether this myogenic process is the same in sheep as in pigs at earlier embryonic stages remains to be investigated.

We found that the immune and hematological systems' development and function were most overrepresented of all physiological systems and functions, accompanied by muscle system development, which was consistent with a microarray developmental analysis in cattle fetuses [21]. Recent discoveries have revealed complex interactions between skeletal muscle and the immune system that regulate muscle regeneration and myogenesis, and many immune molecules, such as tumor necrosis factor-alpha (TNF-α), NF-kB, interleukin (IL)-4, IL-6, IL-10, and leukemia inhibitory factor are involved in muscle cell proliferation and differentiation [33-38]. In most cases, whether perturbation in the same signaling pathways related to myogenesis occurs will determine normal myogenic development or muscle disorders. Therefore, the pathways associated with immune and muscle disorders in this work are valuable for human myopathy at the prenatal stage. The hematological system is also implicated in myogenesis [39-43], which probably reflects the systemic requirement for muscle function during fetal development.

Nervous system development was overrepresented in the last two development stages in the two sheep breeds. Neurons are indispensable for maintaining normal muscle physiological function and also parallel the skeletal muscle in auxology. Comprehensive and close interactions occur between muscles and the nervous system during development [44-46]. Muscle development is regulated by the central nervous system in *Drosophila *and pigs [47,48], and muscle fiber type is dependent on the pattern of innervation of a muscle established due to differential projection patterns between fast and slow motoneurons [49,50]. Animals with different muscle phenotypes undergo diverse innervation patterns during fetal development. Double-muscled cattle have an additional 13-26% increased branching in terminal axons compared to that in normal cattle caused by a real increase in the number of myofibers [51].

We identified several valuable canonical pathways directly associated with muscle development and function, myogenesis, myoblast proliferation, and the cell cycle in muscle at various developmental stages, such as calcium [52], CXCR4 [53-55], and VEGF signaling [56]. Furthermore, other pathways involved in adipose and muscle metabolism, such as inositol metabolism, steroid biosynthesis, EIF2 signaling, glycerolipid metabolism, the glycolysis/gluconeogenesis pathway, and *N*-glycan biosynthesis, are significantly affected during development. Steelman *et al. *suggested that Wnt signaling is a potential downstream target of myostatin for postnatal skeletal muscle growth and hypertrophy in mice [57]. However, we focused on the transcriptome during prenatal stages rather than postnatal stages and found that various canonical pathways are predominant in skeletal muscle development at different stages due to a myostatin mutation in sheep.

### DE genes involved in muscle and adipose development

*STAT3*, a member of the *STAT *family and a cooperator in the Janus kinase pathway, plays a dual role in the regulation of myoblast proliferation and differentiation, but is dependent on interactions with various cofactors [58-62]. We found that *STAT3 *expression was higher in U70 than T70, which suggests that *STAT3 *is most probably associated with the differentiation rather than proliferation of myoblasts. The GO analysis of DE genes between T70 and U70 confirmed our presumption, and muscle fiber hyperplasia was more intense in T70 (Figure 2) as well.

*ALDOA *is responsible for significant activation during the differentiation of chicken primary myoblasts and plays an important role in muscle gene transcription [63,64]. In our study, a higher *ALDOA *expression level in U70 indicated that more myoblasts exited the cell cycle and entered differentiation in U70 than those in T70. The leptin receptor (LEPR), a protein secreted from adipocytes, is responsible for fat mass regulation via leptin in the hypothalamus [65,66]. *LEPR *expression was higher in U70 than in T70. Whether the higher *LEPR *expression at prenatal stages is associated with high postnatal adipose deposition remains to be investigated.

Both hyperplasia and hypertrophy continued in skeletal muscle at 85 d. *FBP2*, which encodes a gluconeogenesis regulatory enzyme that catalyzes the hydrolysis of fructose 1,6-bisphosphate to fructose 6-phosphate and inorganic phosphate, was expressed higher in T85 than in U85. A previous study suggested that FBPase participates in some nuclear processes during the development and regeneration of skeletal muscle [67,68]. Additionally, *PRG3 *expression was also higher in T85. Previous studies have shown that *PRG3 *plays a role in some early aspects of skeletal myogenesis [69-72].

The expression levels of *PIK3R1, TSHR*, and *PON1 *were higher in U85 and U100 than that in T85 or T100. Previous studies have demonstrated that these genes are involved in fat metabolism and adipocyte development [73-76].

PDK4, a gene that was upregulated in T100, is a key regulatory enzyme involved in switching the energy source from glucose to fatty acids in response to physiological conditions. The pyruvate dehydrogenase complex occupies a central and strategic position in muscle intermediary metabolism and is primarily regulated by phosphorylation/dephosphorylation [77]. In addition, *PDK4 *is significantly associated with intramuscular fat and muscle water content [78], indicating that *PDK4 *is involved in meat quality.

TXNRD1 is a variant of thioredoxin reductase, an important selenoprotein that maintains cellular redox balance and regulates several redox-dependent processes during apoptosis, cell proliferation, and differentiation [79-81]. LEF1 is a transcription factor involved in the regulation of myogenesis. Loss of Lef1-mediated repression results in an increased number of cells expressing Pax-7 and Pax-3, suggesting that Wnt signaling via Lef1 acts to regulate the number of premyogenic cells in somites [82]. In our study, *LEF1 *expression was higher in U100 than that in T100, which is consistent with the rapid proliferation of myofibers in U100. Although transcription factor activity is predominantly manifested at the protein level, the difference in *LEF1 *expression level indicated a potential difference of LEF1 between breeds.

*OVAR-DQB1 *(sheep MHC class II) was expressed much higher in T100 and T120, particularly in T120 (Table 7), compared with U 100 and U120. Results from Karpati *et al. *indicated that the class I molecule may be involved in the fusion of myogenic cells during muscle regeneration [83]. Honda and Rostami demonstrated that the expression of class I antigens on muscle cells is not only immunologically modulated but also developmentally regulated, and that these antigens may play a role in cell recognition and interaction during the myogenic fusion process. The presence of the antigens, however, was transitory, and they disappeared as myoblasts fused and differentiated into multinucleate myotubes [84].

CEBPB, an important transcription factor, acts as a indispensable regulator of adipocyte differentiation during adipogenesis [85-87]. We found that *CEBPB *expression was higher in skeletal muscle in T120 than in U120, indicating a key difference in adipogenesis between Texel and Ujumqin sheep. However, which breeds contain more adipose mass than the other at this developmental stage remains to be investigated.

*TRRAP*, which was downregulated in T120, in contrast to that in U120, is vital for embryonic survival and control of the mitotic checkpoint [88-91]. Deletion of *TRRAP *leads to a reduced level of beta-catenin ubiquitination, a lower degradation rate, and accumulation of the beta-catenin protein, whereas *TRRAP *knockdown results in abnormal retention of beta-catenin at chromatin and concomitant hyperactivation of the canonical Wnt pathway [92]. However, the canonical Wnt pathway is downregulated in the absence of myostatin through beta-catenin, whereas the Wnt/calcium pathway is upregulated [57], suggesting that *TRRAP *may negatively regulate skeletal muscle development between the canonical Wnt and the Wnt/calcium pathways through beta-catenin at 120 d. Further efforts are warranted to test the effect of *TRRAP *on skeletal muscle development.

Other than the genes discussed above, we found that *MSTN *expression was lower in Texel than in Ujumqin sheep through the five development stages (Table 8). This result indicated that *MSTN *downregulation contributed to the hyperplasia and hypertrophy of myofibers in Texel sheep through the second half of gestation. Moreover, the genes discussed above are potential myostatin targets for further investigation.

## Conclusions

We demonstrated that fetal myofiber number increased sharply in a pulsed-wave mode at a particular developmental stage and was not augmented evenly across developmental stages in large mammal. The two surges in myofiber hyperplasia occurred at 85 and 120 d in Texel sheep, whereas a single surge appeared at 100 d in Ujumqin sheep during the second half of gestation. A myostatin mutation changed the gene expression profile in prenatal skeletal muscle, particularly disrupting some pivotal signaling pathways governing muscle development and function at some developmental stages, which explains much of the variation in myofiber phenotypes between Texel and Ujumqin sheep. Further studies on the crucial DE genes and signaling pathways involved would be helpful for revealing the mystery of muscle development in mammals.

## Methods

### Animals

All experimental and surgical procedures were approved by the Biological Studies Animal Care and Use Committee, Shanxi Province, Peoples Republic of China. Seventy-eight Ujumqin and 54 Texel ewes were prepared. These purebred female animals involved were selected based on their age (3-5 years old), body weight (50-55 kg), and body size. After these animals were subjected to pre-feeding for 45 days, estrus was synchronized in all ewes using an implanted controlled internal drug release device (Pharmacia & Upjohn Pty Limited, Parramatta City, NSW, Australia) and intramuscular injections of pregnant mare serum gonadotropin (Ningbo Renjian Pharmaceutical Co., Ltd, Zhejiang, China), according to the manufacturers' protocol, followed by artificial insemination using the corresponding breed sire's sperm. Three pregnant ewes from each breed were subject to caesarean section to collect the fetuses at 70, 85, 100, 120, and 135 d of gestation, and then the 12 different anatomic skeletal muscles from each fetus were dissected and weighed (longissimus dorsi, semitendenosus, semimembranosus, gluteus medius, femoral quadriceps, gastrocnemius, serratus ventralis thoracis muscle, biceps femoris, adductor, supraspinatus, infraspinatus, and triceps brachii). Two samples were taken from each muscle at standardized anatomical sites. The first sample (3 g) was dissected and rapidly frozen whole in isopentane chilled over liquid nitrogen for histological examination. The second sample (5 g) was snap-frozen in liquid nitrogen for gene expression analysis.

### Histology analysis

To examine the development of skeletal muscle in both sheep breeds, we measured the number and the diameter of the longissimus dorsi muscle fiber in Ujumqin and Texel sheep during five development stages using hematoxylin and eosin staining. Serial cross sections of 10 μm thickness were cut at -20°C using a cryostat. Photomicrographs of five to eight randomly selected areas were used to estimate the the diameter and the total number of myofibers in the cross sections of muscle examined. Muscle fiber measurements were conducted using DT2000 (V2.0) image analysis software (Nanjing East-image Ltd., Nanjing, China).

The primary IFS antibodies were rabbit-laminin antibody (Sigma, St. Louis, MO, USA) and mouse monoclonal anti-pax7 (Developmental Studies Hybridoma Bank, University of Iowa, Iowa City, IA, USA). The corresponding secondary antibodies were anti-mouse immunoglobulin (ALEXA488) and anti-rabbit immunoglobulin (ALEXA594) (Invitrogen, Carlsbad, CA, USA). Staining was performed according to the manufacturer's instructions. The IFS slides were analyzed using a system of epi-fluorescence microscopes (TE2000U; Nikon, Tokyo, Japan). The satellite cell (SC) nuclei were stained green and the basal lamina, red. Images were subsequently analyzed using Image Pro Plus (v5.1) image analysis software (Media Cybernetics, Inc., Bethesda, MD, USA). The numbers of SCs and myofibers in each section were counted, and the SC concentration within a given area of view was also calculated for each slide.

Data between the two breeds were analyzed using the *t*-test procedure in SPSS 13.0 (SPSS, Inc., Chicago, IL, USA). A p-value of < 0.05 was considered statistically significant.

### Microarray experiment

We made five independent comparisons at the same development stages in both breeds using a *t*-test and detected 207, 115, 90, 151, and 72 DE probes with a greater than twofold change at 70, 85, 100, 120, and 135 d of gestation, respectively. The new specialized and standardized transcriptome-wide sheep microarray (Agilent Technologies) contained 15,008 sheep probes sourced from RefSeq and UniGene. The oligo microarray was 15 K in size. The longissimus dorsi muscles from ovine fetuses were investigated for gene expression. The microarray of each development stage for each breed contained three biological replicates (n = 3) except the T100 team (n = 4). Thus, 31 hybridizations in total were performed. Data were deposited in the NCBI database (GSE23563).

Total RNA was extracted from the frozen longissimus dorsi muscles using an RNeasy Mini Kit with DNase treatment (Qiagen, Valencia, CA, USA) according to the manufacturer's protocol. The total RNA from each individual was labeled with Cy3 fluorescence. The generated targets were mixed and subjected to hybridization with the Sheep Gene Expression Microarray (Agilent Technologies), according to the manufacturer's protocol.

Scanning of the microarrays was performed using a DNA microarray scanner (Agilent Technologies). The scanning parameters were a 5 μm scan resolution, PMT with 100%, and 10% once, respectively. All probe sets whose signals were reported as "absent" during the flag call in all 31 arrays were filtered out, and those from probe sets called "present" on at least one chip were used for further analysis. Scanner output image files were normalized and filtered using Feature Extraction Software v10.5 (Agilent Technologies).

### Statistical analysis of the microarray data

The raw signal intensity data were normalized using the quantile method, and the data were transformed to a base-2 logarithm for further statistical analysis. To explore the difference in gene expression between breeds at each developmental stage, we conducted a comparative analysis with the unpaired unequal variance *t*-test (Welch's *t*-test) method in GeneSpring10.0 (Agilent Technologies). Five *t*-test contrasts were performed independently, based on the premise that no relationships existed between the data at different time points. The thresholds for significance were p < 0.05 and a fold change of twofold or more with FDR < 0.4.

### Gene ontology and functional annotation analysis

Only 1508 of the 15,008 probe set were definitive in the Agilent Sheep Gene Expression Microarray. To further clarify the function of the DE genes in this study, we conducted a BLASTN search for highly homologous human sequences (search setting: query coverage not less than 50%, E value less than 1.00E-100). DAVID Bioinformatics Resources (http://david.abcc.ncifcrf.gov) [93,94] and IPA (http://www.ingenuity.com) [95] were both used for further functional analysis to identify which molecular events or cascades were involved during skeletal muscle development. Significance was expressed as a p-value, which was calculated using the EASE score (p-values < 0.05 were considered significant).

### Quantitative real-time PCR analysis

Total RNA was used to make cDNA using the PrimeScript^® ^1st Strand cDNA Synthesis Kit (Takara Bio, Shiga, Japan). q-Real-time PCR was performed on an ABI 7500 instrument (Applied Biosystems, Foster City, CA, USA) using the Fast EvaGreen^® ^Master Mix (Biotium, Hayward, CA, USA). Thermal cycling consisted of an initial step at 95°C for 10 min followed by 40 cycles at 95°C for 30 s and 62°C for 30 s. Fifteen DE genes identified in the microarray experiment were selected for validation by q-real-time PCR; *RpLP0 *was chosen as the reference gene for normalization of all data because it was expressed more stably [26-29]. Each q-real-time PCR reaction (in 20 μl) contained 10 μl of Fast EvaGreen^® ^Master Mix, 0.5 μl of each primer, 2 μl of normalized template cDNA, and 7 μl water. The q-real-time PCR measurements were performed in triplicate on each cDNA sample (n = 31), and gene expression was quantified relative to *RpLP0 *expression using the 2-ΔΔCt method. Gene expression ratios were normalized to the *RpLP0 *gene in the same sample.

## Competing interests

There were no competing financial interests (political, personal, religious, ideological, academic, intellectual, commercial, or any other) in relation to this manuscript. All authors agreed to publish the manuscript in this journal.

## Authors' contributions

HXR participated in the experimental design, sample collection, data analysis, and drafted the manuscript. LL participated in the experimental design, sample collection, and the microarray experiment. HWS performed the histological analysis and participated in sampling. LYX participated in the microarray data analysis and sample collection. CHW and LZ extracted the total RNA and conducted the qPCR validation. HBL was actively involved in sampling and coordination. WZL provided the experimental environment and coordination. LXD organized the entire experiment and helped draft the manuscript. All authors have read and approved the final manuscript.

## Supplementary Material

Additional file 1 **Quality control and validity of the gene expression microarray**. This file contains the analytic settings using GeneSpring10.0 (Agilent) and the detectable rate of Agilent Sheep Gene Expression Microarray. Click here for file

Additional file 2 **Differentially expressed (DE) genes in skeletal muscle between Texel and Ujumqin sheep at 70 d**. For Additional files 2-6, the *t*-test was employed to identify the DE genes between Texel and Ujumqin sheep at each developmental stage using GeneSpring 10.0 (Agilent). Only those genes with p ≤ 0.05(p-value in Colum B) and an absolute twofold change or more(absolute FoldChange in Colum C) were considered DE genes for further analysis. In the "regulation" column, "up" or "down" indicates that the genes were upregulated or downregulated in Texel fetal sheep, respectively, compared with those in Ujumqin fetal sheep. Click here for file

Additional file 3 **Differentially expressed (DE) genes in skeletal muscle between Texel and Ujumqin sheep at 85 d**. Click here for file

Additional file 4 **Differentially expressed (DE) genes in skeletal muscle between Texel and Ujumqin sheep at 100 d**. Click here for file

Additional file 5 **Differentially expressed (DE) genes in skeletal muscle between Texel and Ujumqin sheep at 120 d**. Click here for file

Additional file 6 **Differentially expressed (DE) genes in skeletal muscle between Texel and Ujumqin sheep at 135 d**. Click here for file

## References

[B1] Hickford JG Forrest RH Zhou H Association between a g+6723G-A SNP in the myostatin gene (MSTN) and carcass traits in New Zealand Texel sheep Journal of animal science 2009 87 6 1853 10.2527/jas.2009-1960 19443859

[B2] Johnson PL Dodds KG Bain WE Greer GJ McLean NJ McLaren RJ Galloway SM van Stijn TC McEwan JC Investigations into the GDF8 g+6723G-A polymorphism in New Zealand Texel sheep Journal of animal science 2009 87 6 1856 1864 10.2527/jas.2008-1508 19251921

[B3] Clop A Marcq F Takeda H Pirottin D Tordoir X Bibe B Bouix J Caiment F Elsen JM Eychenne F A mutation creating a potential illegitimate microRNA target site in the myostatin gene affects muscularity in sheep Nature genetics 2006 38 7 813 818 10.1038/ng1810 16751773

[B4] Johnson PL McEwan JC Dodds KG Purchas RW Blair HT Meat quality traits were unaffected by a quantitative trait locus affecting leg composition traits in Texel sheep Journal of animal science 2005 83 12 2729 2735 16282610 10.2527/2005.83122729x

[B5] Kijas JW McCulloch R Edwards JE Oddy VH Lee SH van der Werf J Evidence for multiple alleles effecting muscling and fatness at the ovine GDF8 locus BMC genetics 2007 8 80 17996073 10.1186/1471-2156-8-80 PMC2212645

[B6] Meng XR Guo J Zhao QJ Ma YH Guan WJ Liu D Di R Qiao HY Na RS [Variation of MSTN gene UTR in eleven sheep breeds] Yi chuan = Hereditas/Zhongguo yi chuan xue hui bian ji 2008 30 1585 1590 19073574 10.3724/sp.j.1005.2008.01585

[B7] Rehfeldt C Fiedler I Stickland NC te Pas MFW HH, Everts ME Number and size of muscle fibres in relation to meat production Muscle Development of Livestock Animals: Physiology, Genetics, and Meat Quality 2004 Wallingford: Oxfordshire: CAB Int 1 37

[B8] Ashmore CR Robinson DW Rattray P Doerr L Biphasic development of muscle fibers in the fetal lamb Experimental neurology 1972 37 2 241 255 10.1016/0014-4886(72)90071-4 4118074

[B9] Maier A McEwan JC Dodds KG Fischman DA Fitzsimons RB Harris AJ Myosin heavy chain composition of single fibres and their origins and distribution in developing fascicles of sheep tibialis cranialis muscles Journal of muscle research and cell motility 1992 13 5 551 572 10.1007/BF01737997 1460083

[B10] Russell RG Oteruelo FT An ultrastructural study of the differentiation of skeletal muscle in the bovine fetus Anatomy and embryology 1981 162 4 403 417 10.1007/BF00301866 7347494

[B11] Wilson SJ McEwan JC Sheard PW Harris AJ Early stages of myogenesis in a large mammal: formation of successive generations of myotubes in sheep tibialis cranialis muscle Journal of muscle research and cell motility 1992 13 5 534 550 10.1007/BF01737996 1460082

[B12] McCoard SA McNabb WC Peterson SW McCutcheon SN Harris PM Muscle growth, cell number, type and morphometry in single and twin fetal lambs during mid to late gestation Reproduction, fertility, and development 2000 12 5-6 319 327 11451023 10.1071/rd99059

[B13] Lee SJ McPherron AC Regulation of myostatin activity and muscle growth Proceedings of the National Academy of Sciences of the United States of America 2001 98 16 9306 9311 10.1073/pnas.151270098 11459935 PMC55416

[B14] McPherron AC Lee SJ Double muscling in cattle due to mutations in the myostatin gene Proceedings of the National Academy of Sciences of the United States of America 1997 94 23 12457 12461 10.1073/pnas.94.23.12457 9356471 PMC24998

[B15] Mosher DS Quignon P Bustamante CD Sutter NB Mellersh CS Parker HG Ostrander EA A mutation in the myostatin gene increases muscle mass and enhances racing performance in heterozygote dogs PLoS genetics 2007 3 5 e79 10.1371/journal.pgen.0030079 17530926 PMC1877876

[B16] Schuelke M Wagner KR Stolz LE Hubner C Riebel T Komen W Braun T Tobin JF Lee SJ Myostatin mutation associated with gross muscle hypertrophy in a child The New England journal of medicine 2004 350 26 2682 2688 10.1056/NEJMoa040933 15215484

[B17] Grobet L Martin LJ Poncelet D Pirottin D Brouwers B Riquet J Schoeberlein A Dunner S Menissier F Massabanda J A deletion in the bovine myostatin gene causes the double-muscled phenotype in cattle Nature genetics 1997 17 1 71 74 10.1038/ng0997-71 9288100

[B18] Kambadur R Sharma M Smith TP Bass JJ Mutations in myostatin (GDF8) in double-muscled Belgian Blue and Piedmontese cattle Genome research 1997 7 9 910 916 9314496 10.1101/gr.7.9.910

[B19] Cassar-Malek I Passelaigue F Bernard C Leger J Hocquette JF Target genes of myostatin loss-of-function in muscles of late bovine fetuses BMC genomics 2007 8 63 10.1186/1471-2164-8-63 17331240 PMC1831773

[B20] Lehnert SA Reverter A Byrne KA Wang Y Nattrass GS Hudson NJ Greenwood PL Gene expression studies of developing bovine longissimus muscle from two different beef cattle breeds BMC developmental biology 2007 7 95 10.1186/1471-213X-7-95 17697390 PMC2031903

[B21] Hudson NJ Reverter A Dalrymple BP A differential wiring analysis of expression data correctly identifies the gene containing the causal mutation PLoS computational biology 2009 5 5 e1000382 10.1371/journal.pcbi.1000382 19412532 PMC2671163

[B22] Hudson NJ Reverter A Wang Y Greenwood PL Dalrymple BP Inferring the transcriptional landscape of bovine skeletal muscle by integrating co-expression networks PloS one 2009 4 10 e7249 10.1371/journal.pone.0007249 19794913 PMC2749936

[B23] Biressi S Tagliafico E Lamorte G Monteverde S Tenedini E Roncaglia E Ferrari S Ferrari S Cusella-De Angelis MG Tajbakhsh S Intrinsic phenotypic diversity of embryonic and fetal myoblasts is revealed by genome-wide gene expression analysis on purified cells Developmental biology 2007 304 2 633 651 10.1016/j.ydbio.2007.01.016 17292343

[B24] Swatland HJ Cassens RG Inhibition of muscle growth in foetal sheep The Journal of Agricultural Science 1973 80 503 509

[B25] Kijas JW Menzies M Ingham A Sequence diversity and rates of molecular evolution between sheep and cattle genes Animal genetics 2006 37 2 171 174 10.1111/j.1365-2052.2005.01399.x 16573533

[B26] Fleming-Waddell JN Wilson LM Olbricht GR Vuocolo T Byrne K Craig BA Tellam RL Cockett NE Bidwell CA Analysis of gene expression during the onset of muscle hypertrophy in callipyge lambs Animal genetics 2007 38 1 28 36 10.1111/j.1365-2052.2006.01562.x 17257185

[B27] White JD Vuocolo T McDonagh M Grounds MD Harper GS Cockett NE Tellam R Analysis of the callipyge phenotype through skeletal muscle development; association of Dlk1 with muscle precursor cells Differentiation; research in biological diversity 2008 76 3 283 298 17697128 10.1111/j.1432-0436.2007.00208.x

[B28] Vuocolo T Byrne K White J McWilliam S Reverter A Cockett NE Tellam RL Identification of a gene network contributing to hypertrophy in callipyge skeletal muscle Physiological genomics 2007 28 3 253 272 17077277 10.1152/physiolgenomics.00121.2006

[B29] Laborda J 36B4 cDNA used as an estradiol-independent mRNA control is the cDNA for human acidic ribosomal phosphoprotein PO Nucleic acids research 1991 19 14 3998 10.1093/nar/19.14.3998 1861990 PMC328497

[B30] Relaix F Rocancourt D Mansouri A Buckingham M A Pax3/Pax7-dependent population of skeletal muscle progenitor cells Nature 2005 435 7044 948 953 10.1038/nature03594 15843801

[B31] Seale P Sabourin LA Girgis-Gabardo A Mansouri A Gruss P Rudnicki MA Pax7 is required for the specification of myogenic satellite cells Cell 2000 102 6 777 786 10.1016/S0092-8674(00)00066-0 11030621

[B32] Cagnazzo M te Pas MF Priem J de Wit AA Pool MH Davoli R Russo V Comparison of prenatal muscle tissue expression profiles of two pig breeds differing in muscle characteristics Journal of animal science 2006 84 1 1 10 16361485 10.2527/2006.8411

[B33] Tidball JG Villalta SA Regulatory interactions between muscle and the immune system during muscle regeneration American journal of physiology-Regulatory integrative and comparative physiology 2010 298 R1173 R1187 10.1152/ajpregu.00735.2009 20219869 PMC2867520

[B34] Wang X Wu H Zhang Z Liu S Yang J Chen X Fan M Effects of Interleukin-6, Leukemia Inhibitory Factor, and Ciliary Neurotrophic Factor on the Proliferation and Differentiation of Adult Human Myoblasts Cellular and molecular neurobiology 2008 28 1 113 124 10.1007/s10571-007-9247-9 PMC11515048 18240017

[B35] Okazaki S Kawai H Arii Y Yamaguchi H Saito S Effects of calcitonin gene-related peptide and interleukin 6 on myoblast differentiation Cell Proliferation 1996 29 4 173 182 10.1111/j.1365-2184.1996.tb00104.x 8695746

[B36] Austin L Bower JJ Bennett TM Lynch GS Kapsa R White JD W B Gregorevic P Byrne E Leukemia inhibitory factor ameliorates muscle fiber degeneration in the mdx mouse Muscle & nerve 2000 23 11 1700 1705 10.1002/1097-4598(200011)23:11<1700::AID-MUS5>3.0.CO;2-W 11054748

[B37] Jo C Kim H Jo I Choi I Jung S-C Kim J Kim SS Jo SA Leukemia inhibitory factor blocks early differentiation of skeletal muscle cells by activating ERK Biochimica et Biophysica Acta, Molecular Cell Research 2005 1743 3 187 197 10.1016/j.bbamcr.2004.11.002 15843032

[B38] Spangenburg EE Booth FW Multiple signaling pathways mediate LIF-induced skeletal muscle satellite cell proliferation American journal of physiology 2002 283 C204 C211 12055089 10.1152/ajpcell.00574.2001

[B39] De Angelis L Berghella L Coletta M Lattanzi L Zanchi M Cusella-De Angelis MG Ponzetto C Cossu G Skeletal myogenic progenitors originating from embryonic dorsal aorta coexpress endothelial and myogenic markers and contribute to postnatal muscle growth and regeneration The Journal of cell biology 1999 147 4 869 878 10.1083/jcb.147.4.869 10562287 PMC2156164

[B40] Jay KE Gallacher L Bhatia M Emergence of muscle and neural hematopoiesis in humans Blood 2002 100 9 3193 3202 10.1182/blood-2002-02-0502 12384417

[B41] Ferrari G Cusella-De Angelis G Coletta M Paolucci E Stornaiuolo A Cossu G Mavilio F Muscle regeneration by bone marrow-derived myogenic progenitors Science (New York, NY) 1998 279 5356 1528 1530 10.1126/science.279.5356.1528 9488650

[B42] Takahashi A Kureishi Y Yang J Luo Z Guo K Mukhopadhyay D Ivashchenko Y Branellec D Walsh K Myogenic Akt signaling regulates blood vessel recruitment during myofiber growth Molecular and cellular biology 2002 22 13 4803 4814 10.1128/MCB.22.13.4803-4814.2002 12052887 PMC133891

[B43] Gang EJ Jeong JA Hong SH Hwang SH Kim SW Yang IH Ahn C Han H Kim H Skeletal myogenic differentiation of mesenchymal stem cells isolated from human umbilical cord blood Stem cells (Dayton, Ohio) 2004 22 4 617 624 10.1634/stemcells.22-4-617 15277707

[B44] Bayline RJ Duch C Levine RB Nerve-muscle interactions regulate motor terminal growth and myoblast distribution during muscle development Developmental biology 2001 231 2 348 363 10.1006/dbio.2001.0158 11237464

[B45] Fernandes JJ Keshishian H Nerve-muscle interactions during flight muscle development in Drosophila Development (Cambridge, England) 1998 125 9 1769 1779 10.1242/dev.125.9.1769 9521914

[B46] Sohal GS Sixth Annual Stuart Reiner Memorial Lecture: embryonic development of nerve and muscle Muscle & nerve 1995 18 1 2 14 10.1002/mus.880180103 7799994

[B47] Campion DR Richardson RL Kraeling RR Reagan JO Regulation of skeletal muscle development by the central nervous system in the fetal pig Growth 1978 42 2 189 204 680581

[B48] Lewis JO Crews ST Genetic analysis of the Drosophila single-minded gene reveals a central nervous system influence on muscle development Mechanisms of development 1994 48 2 81 91 10.1016/0925-4773(94)90018-3 7873405

[B49] McLennan IS The development of the pattern of innervation in chicken hindlimb muscles: evidence for specification of nerve-muscle connections Developmental biology 1983 97 1 229 238 10.1016/0012-1606(83)90080-5 6188640

[B50] Rafuse VF Milner LD Landmesser LT Selective innervation of fast and slow muscle regions during early chick neuromuscular development The Journal of Neuroscience 1996 16 21 6864 6877 8824325 10.1523/JNEUROSCI.16-21-06864.1996 PMC6579250

[B51] Swatland HJ Cassens RG The role of innervation in muscle development and function Journal of animal science 1974 38 5 1092 1102 4274989 10.2527/jas1974.3851092x

[B52] Gailly P New aspects of calcium signaling in skeletal muscle cells: implications in Duchenne muscular dystrophy Biochimica et biophysica acta 2002 1600 1-2 38 44 12445457 10.1016/s1570-9639(02)00442-9

[B53] Odemis V Lamp E Pezeshki G Moepps B Schilling K Gierschik P Littman DR Engele J Mice deficient in the chemokine receptor CXCR4 exhibit impaired limb innervation and myogenesis Molecular and cellular neurosciences 2005 30 4 494 505 10.1016/j.mcn.2005.07.019 16198599

[B54] Vasyutina E Stebler J Brand-Saberi B Schulz S Raz E Birchmeier C CXCR4 and Gab1 cooperate to control the development of migrating muscle progenitor cells Genes & development 2005 19 18 2187 2198 10.1101/gad.346205 16166380 PMC1221889

[B55] Ratajczak MZ Majka M Kucia M Drukala J Pietrzkowski Z Peiper S Janowska-Wieczorek A Expression of functional CXCR4 by muscle satellite cells and secretion of SDF-1 by muscle-derived fibroblasts is associated with the presence of both muscle progenitors in bone marrow and hematopoietic stem/progenitor cells in muscles Stem cells (Dayton, Ohio) 2003 21 3 363 371 10.1634/stemcells.21-3-363 12743331

[B56] Ouchi N Shibata R Walsh K AMP-activated protein kinase signaling stimulates VEGF expression and angiogenesis in skeletal muscle Circulation research 2005 96 8 838 846 10.1161/01.RES.0000163633.10240.3b 15790954

[B57] Steelman CA Recknor JC Nettleton D Reecy JM Transcriptional profiling of myostatin-knockout mice implicates Wnt signaling in postnatal skeletal muscle growth and hypertrophy The FASEB Journal 2006 20 3 580 582 16423875 10.1096/fj.05-5125fje

[B58] Sun L Ma K Wang H Xiao F Gao Y Zhang W Wang K Gao X Ip N Wu Z JAK1-STAT1-STAT3, a key pathway promoting proliferation and preventing premature differentiation of myoblasts The Journal of cell biology 2007 179 1 129 138 10.1083/jcb.200703184 17908914 PMC2064742

[B59] Snyder M Huang XY Zhang JJ Identification of novel direct Stat3 target genes for control of growth and differentiation The Journal of biological chemistry 2008 283 7 3791 3798 18065416 10.1074/jbc.M706976200

[B60] Wang K Wang C Xiao F Wang H Wu Z JAK2/STAT2/STAT3 are required for myogenic differentiation The Journal of biological chemistry 2008 283 49 34029 34036 10.1074/jbc.M803012200 18835816 PMC2662224

[B61] Kataoka Y Matsumura I Ezoe S Nakata S Takigawa E Sato Y Kawasaki A Yokota T Nakajima K Felsani A Reciprocal inhibition between MyoD and STAT3 in the regulation of growth and differentiation of myoblasts The Journal of biological chemistry 2003 278 45 44178 44187 10.1074/jbc.M304884200 12947115

[B62] Megeney LA Perry RL LeCouter JE Rudnicki MA bFGF and LIF signaling activates STAT3 in proliferating myoblasts Developmental genetics 1996 19 2 139 145 10.1002/(SICI)1520-6408(1996)19:2<139::AID-DVG5>3.0.CO;2-A 8900046

[B63] Hidaka K Yamamoto I Arai Y Mukai T The MEF-3 motif is required for MEF-2-mediated skeletal muscle-specific induction of the rat aldolase A gene Molecular and cellular biology 1993 13 10 6469 6478 8413246 10.1128/mcb.13.10.6469-6478.1993 PMC364706

[B64] Walsh TP Winzor DJ Clarke FM Masters CJ Morton DJ Binding of aldolase to actin-containing filaments. Evidence of interaction with the regulatory proteins of skeletal muscle The Biochemical journal 1980 186 1 89 98 6892770 10.1042/bj1860089 PMC1161506

[B65] Kielar D Clark JS Ciechanowicz A Kurzawski G Sulikowski T Naruszewicz M Leptin receptor isoforms expressed in human adipose tissue Metabolism: clinical and experimental 1998 47 7 844 847 10.1016/s0026-0495(98)90124-x 9667233

[B66] Mammes O Aubert R Betoulle D Pean F Herbeth B Visvikis S Siest G Fumeron F LEPR gene polymorphisms: associations with overweight, fat mass and response to diet in women European journal of clinical investigation 2001 31 5 398 404 10.1046/j.1365-2362.2001.00843.x 11380591

[B67] Gizak A Wrobel E Moraczewski J Dzugaj A Changes in subcellular localization of fructose 1,6-bisphosphatase during differentiation of isolated muscle satellite cells FEBS letters 2006 580 17 4042 4046 10.1016/j.febslet.2006.06.042 16814784

[B68] Gizak A Maciaszczyk E Dzugaj A Eschrich K Rakus D Evolutionary conserved N-terminal region of human muscle fructose 1,6-bisphosphatase regulates its activity and the interaction with aldolase Proteins 2008 72 1 209 216 10.1002/prot.21909 18214967

[B69] Carrino DA Oron U Pechak DG Caplan AI Reinitiation of chondroitin sulphate proteoglycan synthesis in regenerating skeletal muscle Development (Cambridge, England) 1988 103 4 641 656 10.1242/dev.103.4.641 3248520

[B70] Miller RR Rao JS Burton WV Festoff BW Proteoglycan synthesis by clonal skeletal muscle cells during in vitro myogenesis: differences detected in the types and patterns from primary cultures International Journal of Developmental Neuroscience 1991 9 3 259 267 10.1016/0736-5748(91)90046-O 1927582

[B71] Miller RR Rao JS Festoff BW Proteoglycan synthesis by primary chick skeletal muscle during in vitro myogenesis Journal of cellular physiology 1987 133 2 258 266 10.1002/jcp.1041330209 3680389

[B72] Larrain J Alvarez J Hassell JR Brandan E Expression of perlecan, a proteoglycan that binds myogenic inhibitory basic fibroblast growth factor, is down regulated during skeletal muscle differentiation Experimental cell research 1997 234 2 405 412 10.1006/excr.1997.3648 9260911

[B73] Zafiropoulos A Linardakis M Jansen EH Tsatsakis AM Kafatos A Tzanakakis GN Paraoxonase 1 R/Q alleles are associated with differential accumulation of saturated versus 20:5n3 fatty acid in human adipose tissue Journal of lipid research 10.1194/jlr.P004960 PMC2882743 20133274

[B74] Nakao C Itoh TJ Hotani H Mori N Modulation of the stathmin-like microtubule destabilizing activity of RB3, a neuron-specific member of the SCG10 family, by its N-terminal domain The Journal of biological chemistry 2004 279 22 23014 23021 10.1074/jbc.M313693200 15039434

[B75] Jamshidi Y Snieder H Wang X Pavitt MJ Spector TD Carter ND O'Dell SD Phosphatidylinositol 3-kinase p85alpha regulatory subunit gene PIK3R1 haplotype is associated with body fat and serum leptin in a female twin population Diabetologia 2006 49 11 2659 2667 10.1007/s00125-006-0388-z 17016694 PMC1626353

[B76] Elgadi A Zemack H Marcus C Norgren S Tissue-specific knockout of TSHr in white adipose tissue increases adipocyte size and decreases TSH-induced lipolysis Biochemical and biophysical research communications 393 3 526 530 10.1016/j.bbrc.2010.02.042 20152797

[B77] Abbot EL McCormack JG Reynet C Hassall DG Buchan KW Yeaman SJ Diverging regulation of pyruvate dehydrogenase kinase isoform gene expression in cultured human muscle cells The FEBS journal 2005 272 12 3004 3014 10.1111/j.1742-4658.2005.04713.x 15955060

[B78] Lan J Lei MG Zhang YB Wang JH Feng XT Xu DQ Gui JF Xiong YZ Characterization of the porcine differentially expressed PDK4 gene and association with meat quality Molecular biology reports 2009 36 7 2003 2010 10.1007/s11033-008-9411-4 19051057

[B79] Arner ES Holmgren A Physiological functions of thioredoxin and thioredoxin reductase European journal of biochemistry/FEBS 2000 267 20 6102 6109 10.1046/j.1432-1327.2000.01701.x 11012661

[B80] Nakamura H Nakamura K Yodoi J Redox regulation of cellular activation Annual review of immunology 1997 15 351 369 10.1146/annurev.immunol.15.1.351 9143692

[B81] Nordberg J Arner ES Reactive oxygen species, antioxidants, and the mammalian thioredoxin system Free radical biology & medicine 2001 31 11 1287 1312 10.1016/S0891-5849(01)00724-9 11728801

[B82] Abu-Elmagd M Robson L Sweetman D Hadley J Francis-West P Munsterberg A Wnt/Lef1 signaling acts via Pitx2 to regulate somite myogenesis Developmental biology 337 2 211 219 10.1016/j.ydbio.2009.10.023 19850024

[B83] Karpati G Pouliot Y Carpenter S Expression of immunoreactive major histocompatibility complex products in human skeletal muscles Annals of neurology 1988 23 1 64 72 10.1002/ana.410230111 3278673

[B84] Honda H Rostami A Expression of major histocompatibility complex class I antigens in rat muscle cultures: the possible developmental role in myogenesis Proceedings of the National Academy of Sciences of the United States of America 1989 86 18 7007 7011 10.1073/pnas.86.18.7007 2571148 PMC297981

[B85] Urs S Venkatesh D Tang Y Henderson T Yang X Friesel RE Rosen CJ Liaw L Sprouty1 is a critical regulatory switch of mesenchymal stem cell lineage allocation The FASEB Journal 24 9 3264 3273 10.1096/fj.10-155127 PMC2923355 20410440

[B86] Tang QQ Otto TC Lane MD CCAAT/enhancer-binding protein beta is required for mitotic clonal expansion during adipogenesis Proceedings of the National Academy of Sciences of the United States of America 2003 100 3 850 855 10.1073/pnas.0337434100 12525691 PMC298690

[B87] Zhang JW Tang QQ Vinson C Lane MD Dominant-negative C/EBP disrupts mitotic clonal expansion and differentiation of 3T3-L1 preadipocytes Proceedings of the National Academy of Sciences of the United States of America 2004 101 1 43 47 14688407 10.1073/pnas.0307229101 PMC314135

[B88] DeRan M Pulvino M Greene E Su C Zhao J Transcriptional activation of histone genes requires NPAT-dependent recruitment of TRRAP-Tip60 complex to histone promoters during the G1/S phase transition Molecular and cellular biology 2008 28 1 435 447 10.1128/MCB.00607-07 17967892 PMC2223310

[B89] Herceg Z Hulla W Gell D Cuenin C Lleonart M Jackson S Wang ZQ Disruption of Trrap causes early embryonic lethality and defects in cell cycle progression Nature genetics 2001 29 2 206 211 10.1038/ng725 11544477

[B90] Bouchard C Dittrich O Kiermaier A Dohmann K Menkel A Eilers M Luscher B Regulation of cyclin D2 gene expression by the Myc/Max/Mad network: Myc-dependent TRRAP recruitment and histone acetylation at the cyclin D2 promoter Genes & development 2001 15 16 2042 2047 10.1101/gad.907901 11511535 PMC312761

[B91] Li H Cuenin C Murr R Wang ZQ Herceg Z HAT cofactor Trrap regulates the mitotic checkpoint by modulation of Mad1 and Mad2 expression The EMBO journal 2004 23 24 4824 4834 10.1038/sj.emboj.7600479 15549134 PMC535091

[B92] Finkbeiner MG Sawan C Ouzounova M Murr R Herceg Z HAT cofactor TRRAP mediates beta-catenin ubiquitination on the chromatin and the regulation of the canonical Wnt pathway Cell cycle (Georgetown, Tex) 2008 7 24 3908 3914 10.4161/cc.7.24.7354 19066453

[B93] Dennis G Jr Sherman BT Hosack DA Yang J Gao W Lane HC Lempicki RA DAVID: Database for Annotation, Visualization, and Integrated Discovery Genome biology 2003 4 5 P3 10.1186/gb-2003-4-5-p3 12734009 PMC193660

[B94] Huang da W Sherman BT Lempicki RA Systematic and integrative analysis of large gene lists using DAVID bioinformatics resources Nature protocols 2009 4 1 44 57 19131956 10.1038/nprot.2008.211

[B95] Ingenuity Pathway Analysis http://www.ingenuity.com/

