# Supplementary material for: Histological and transcriptome-wide level characteristics of fetal myofiber hyperplasia during the second half of gestation in Texel and Ujumqin sheep
Source: BMC Genomics. 2011 Aug 14;12:411. doi: 10.1186/1471-2164-12-411 (PMC3173453; doi:10.1186/1471-2164-12-411)
Supplement: Additional file 1 — Quality control and validity of the gene expression microarray. This file contains the analytic settings using GeneSpring10.0 (Agilent) and the detectable rate of Agilent Sheep Gene Expression Microarray. [file 1471-2164-12-411-S1.DOC]

Additional file 1.

Quality control and validity of Sheep Gene Expression Microarray

Figure S1. Settings of analysis using GeneSpring10.0 (Agilent).

Table S1. Detectable rate of Agilent Sheep Gene Expression Microarray.

| **Normalized File Name** | **No. of Sample /Cy3** | **CV（%）*** | **Detectable Rate*** |
| --- | --- | --- | --- |
| 10054_1_1 | 1 | 11.22 | 90.43% |
| 10054_2_1 | 10 | 6.59 | 87.71% |
| 10054_2_2 | 11 | 8.20 | 84.01% |
| 10054_2_3 | 12 | 9.18 | 88.48% |
| 10054_2_4 | 14 | 9.51 | 89.23% |
| 10055_1_1 | 15 | 6.31 | 89.43% |
| 10055_1_2 | 16 | 7.43 | 91.12% |
| 10055_1_3 | 17 | 6.23 | 90.07% |
| 10055_1_4 | 19 | 7.81 | 89.29% |
| 10055_2_1 | 20 | 7.17 | 85.95% |
| 10055_2_2 | 21 | 6.03 | 85.37% |
| 10055_2_3 | 22 | 7.58 | 88.89% |
| 10055_2_4 | 24 | 7.06 | 89.66% |
| 10064_1_1 | 25 | 7.63 | 90.94% |
| 10064_1_2 | 26 | 7.46 | 89.29% |
| 10064_2_2 | 34 | 8.57 | 89.58% |
| 10064_2_3 | 36 | 6.85 | 89.53% |
| 10064_2_4 | 39 | 7.62 | 89.21% |
| 10065_1_1 | 40 | 6.72 | 89.07% |
| 10065_1_2 | 41 | 6.94 | 88.98% |
| 10065_1_3 | 42 | 6.53 | 88.23% |
| 10065_1_4 | 43 | 7.36 | 88.43% |
| 10065_2_1 | 44 | 6.97 | 86.92% |
| 10065_2_2 | 45 | 6.79 | 85.78% |
| 10065_2_3 | 46 | 5.58 | 88.39% |
| 10065_2_4 | 47 | 9.41 | 81.53% |
| 10066_1_1 | 48 | 9.86 | 86.05% |
| 10066_1_2 | 49 | 7.27 | 86.09% |
| 10066_2_2 | 53 | 7.94 | 83.95% |
| 10066_2_3 | 54 | 7.87 | 85.57% |
| 10066_2_4 | 55 | 7.26 | 85.61% |

*Coefficient of Variation（CV）:

CV = Standard deviation(SD)/Mean ×100%. In Agilent expression microarray experiments,the robustness of technique and microarray is evaluated by CV of repeat probe spots(n=10). According to the various microarray, the mumber od repeat probe spots varies from 20 to 100.The propositional Quality control standard by Agilent: CV < 15%.

*Algorithm for Detectable Rate:

Detectable Rate = count of detectable spots / sum of all spots × 100%, that is the ratio of the expressed transcripts. Signals of the “Absent” flag call were excluded for estimation of the Detectable Rate.
